# *In vitro* inhibitory effects of plant-based foods and their combinations on intestinal α-glucosidase and pancreatic α-amylase

**DOI:** 10.1186/1472-6882-12-110

**Published:** 2012-07-31

**Authors:** Sirichai Adisakwattana, Thanyachanok Ruengsamran, Patcharaporn Kampa, Weerachat Sompong

**Affiliations:** 1 The Medical Food Research and Development Center, Department of Nutrition and Dietetics, Faculty of Allied Health Sciences, Chulalongkorn University, Bangkok 10330, Thailand; 2 Research Group of Herbal Medicine for Prevention and Therapeutic of Metabolic diseases, Chulalongkorn University, Bangkok 10330, Thailand; 3 Program in Nutrition and Dietetics, Department of Nutrition and Dietetics, Faculty of Allied Health Sciences, Chulalongkorn University, Bangkok 10330, Thailand

## Abstract

**Background:**

Plant-based foods have been used in traditional health systems to treat diabetes mellitus. The successful prevention of the onset of diabetes consists in controlling postprandial hyperglycemia by the inhibition of α-glucosidase and pancreatic α-amylase activities, resulting in aggressive delay of carbohydrate digestion to absorbable monosaccharide. In this study, five plant-based foods were investigated for intestinal α-glucosidase and pancreatic α-amylase. The combined inhibitory effects of plant-based foods were also evaluated. Preliminary phytochemical analysis of plant-based foods was performed in order to determine the total phenolic and flavonoid content.

**Methods:**

The dried plants of *Hibiscus sabdariffa* (Roselle), *Chrysanthemum indicum* (chrysanthemum), *Morus alba* (mulberry), *Aegle marmelos* (bael), and *Clitoria ternatea* (butterfly pea) were extracted with distilled water and dried using spray drying process. The dried extracts were determined for the total phenolic and flavonoid content by using Folin-Ciocateu’s reagent and AlCl_3_ assay, respectively. The dried extract of plant-based food was further quantified with respect to intestinal α-glucosidase (maltase and sucrase) inhibition and pancreatic α-amylase inhibition by glucose oxidase method and dinitrosalicylic (DNS) reagent, respectively.

**Results:**

The phytochemical analysis revealed that the total phenolic content of the dried extracts were in the range of 230.3-460.0 mg gallic acid equivalent/g dried extract. The dried extracts contained flavonoid in the range of 50.3-114.8 mg quercetin equivalent/g dried extract. It was noted that the IC_50_ values of chrysanthemum, mulberry and butterfly pea extracts were 4.24±0.12 mg/ml, 0.59±0.06 mg/ml, and 3.15±0.19 mg/ml, respectively. In addition, the IC_50_ values of chrysanthemum, mulberry and butterfly pea extracts against intestinal sucrase were 3.85±0.41 mg/ml, 0.94±0.11 mg/ml, and 4.41±0.15 mg/ml, respectively. Furthermore, the IC_50_ values of roselle and butterfly pea extracts against pancreatic α-amylase occurred at concentration of 3.52±0.15 mg/ml and 4.05±0.32 mg/ml, respectively. Combining roselle, chrysanthemum, and butterfly pea extracts with mulberry extract showed additive interaction on intestinal maltase inhibition. The results also demonstrated that the combination of chrysanthemum, mulberry, or bael extracts together with roselle extract produced synergistic inhibition, whereas roselle extract showed additive inhibition when combined with butterfly pea extract against pancreatic α-amylase.

**Conclusions:**

The present study presents data from five plant-based foods evaluating the intestinal α-glucosidase and pancreatic α-amylase inhibitory activities and their additive and synergistic interactions. These results could be useful for developing functional foods by combination of plant-based foods for treatment and prevention of diabetes mellitus.

## Background

Diabetes mellitus is an endocrine and metabolic disorder characterized by chronic hyperglycemia, dyslipidemia, and protein metabolism that result from defects in both regulations of insulin secretion and/or insulin action. There has been a dramatic increase in the number of diabetic patients worldwide because of changes in lifestyle and diet. Consumption of high-carbohydrate diets causes elevated postprandial hyperglycemia that can progress to full symptomatic type 2 diabetes [1]. Current therapeutic strategy for the control of postprandial hyperglycemia is the inhibition of α-glucosidase and α-amylase, resulting in aggressive delay of carbohydrate digestion to absorbable monosaccharide [2]. α-Glucosidase inhibitor has been recognized as a therapeutic approach for modulation of postprandial hyperglycemia, which is the earliest metabolic defect to occur in type 2 diabetes. Current evidence supports the claim that the known α-glucosidase inhibitors such as acarbose and voglibose potentially reduce the progression of diabetes as well as micro- and macrovascular complications including diabetic retinopathy, nephropathy, and neuropathy [3]. However, it has been reported that α-glucosidase and pancreatic α-amylase inhibitors are associated with gastrointestinal side effects such as abdominal pain, flatulence, meteorism, and diarrhea in the diabetic patients [4]. Thus, efforts have been directed at investigating intestinal α-glucosidase and pancreatic α-amylase inhibitors from plant-based foods that are largely free of major undesirable side effects.

Many plant-based foods are good sources of unique phytochemical compounds such as polyphenols and flavonoids. Recent studies have shown that plant-based foods containing high total polyphenolic compounds and flavonoids yield can be linked to intestinal α-glucosidase and pancreatic α-amylase inhibitory activities *in vitro*[5-8]. However, the therapeutic approaches from administration of single plant-based food may be not adequate to assess the delay of carbohydrate digestion. Many scientists have investigated the combination of different plant-based foods containing various phytochemicals that exhibit additive and synergistic interaction in antidiabetic and antioxidant properties that exert positive health-promoting effects, leading to the development of functional foods [9,10]. Hence, we are particularly interested in investigating the inhibitory effect of these plant-based foods and their interactions on the intestinal α-glucosidase and pancreatic α-amylase. For this purpose, a total of 5 plant-based foods were selected, including butterfly pea, mulberry, roselle, bael, and chrysanthemum, which are consumed daily as part of a main meal and beverages in Southeast Asia. Actually, these edible plants are commonly consumed in the form of water extract. Moreover, it is clear that soluble phenolic and flavonoid compounds can be extracted using water. Therefore, total phenolic and flavonoid content in all plant-based foods were also evaluated.

## Methods

### Chemical

Folin-Ciocateu’s reagent, quercetin, gallic acid, rat intestinal acetone powder, porcine pancreatic α-amylase, glucose oxidase kits and 3,5-dinitrosalicylic acid were purchased from Sigma-Aldrich Co. (St. Louis, MO, USA). All other chemical reagents used in this study were of analytical grade.

### Collection and extraction

The plants materials were collected from the local market in Bangkok, Thailand (Table 1) The extraction of the plants was performed according to a previous method [11]. The dried plant materials were subjected to size reduction to a coarse powder by using a dry grinder. The dried plants (200 g) were extracted with distilled water (1,000 ml) at 90°C for 2 h. The samples were filtered through Whatman 70 mm filter paper. The solution was then centrifuged at 8,000 rpm for 10 min. The aqueous solution was dried using a spray dryer SD-100 (Eyela world, Tokyo Rikakikai Co., LTD, Japan). The spray drying conditions used in the study were inlet temperature (164–168°C), outlet temperature (71–78°C), blower (0.70-0.79 m^3^/min) and atomizing (80–90 kPa). 

**Table 1:** The list of plant-based foods was used of this study

**Plant samples**			
**Common name**	**Scientific name**	**Family**	**Used part**
Roselle	*Hibiscus sabdariffa*	Malvaceae	Flower
Chrysanthemum	*Chrysanthemum indicum*	Compositae	Flower
Mulberry	*Morus alba*	Moraceae	Leaves
Bael	*Aegle marmelos*	Rutaceae	Fruit
Butterfly pea	*Clitoria ternatea*	Leguminosae	Flower

### Determination of total phenolic content

Total phenolic content of extracts was performed according to a previous method [11]. The dried extract (0.5 mg) was dissolved in distilled w (1 ml). The sample solution (50 μl) was mixed with 50 μl of Folin-Ciocateu’s reagent followed by 50 μl of Na_2_CO_3_ (10% w/v). After incubation at 30°C for 60 min, the absorbance was then measured at 760 nm using a microplate reader (BioTek Instruments, Inc., USA). Total phenolic content was calculated from a calibration curve using gallic acid as a standard. The results were expressed as milligram gallic acid equivalent/gram dry weight of extract.

### Determination of flavonoid content

Estimation of flavonoid content in the dried extracts was done according to a previous method [11]. The dried extract (0.5 mg) was dissolved in 80% ethanol (1 ml). The sample solution (50 μl) was added to 10 μl of AlCl_3_ solution (10% w/v) and 10 μl of 1 M sodium acetate in absolute ethanol (150 μl). After incubation at 30°C for 30 min, the absorbance was measured immediately at 430 nm. The estimation of flavonoid content was calculated from a calibration curve using quercetin as a standard. The results were expressed as milligram quercetin equivalent/gram dry weight of extract.

### Intestinal α-glucosidase inhibitory activity

The assessment of intestinal α-glucosidase inhibitory activity was based on the modified method previously described [12]. Briefly, 100 mg of rat intestinal acetone powder was homogenized in 3 ml of 0.9% NaCl solution. The solution was centrifuged at 12,000 *g* for 30 min and then subjected to assay. The crude enzyme solution (as maltase assay, 10 μl; as sucrase assay, 30 μl) was incubated with 30 μl maltose (86 mM) or 40 μl sucrose (400 mM), 10 μl of the extract at various concentrations, followed by the addition of 0.1 M phosphate buffer, pH 6.9 to give a final volume of 100 μl. The reaction was incubated at 37°C for 30 min (maltase assay) or 60 min (sucrase assay). Thereafter, the mixtures were suspended in boiling water for 10 min to stop the reaction. The concentrations of glucose released from the reaction mixtures were determined by glucose oxidase method with absorbance at a wavelength of 450 nm. Intestinal α-glucosidase inhibitory activity was expressed as percentage inhibition using the following formula.

(1) $$\%\text{Inhibition }=\frac{Abs_{\mathrm{Control}}-Abs_{\mathrm{Sample}}\;}{Abs_{\mathrm{Control}}}\times 100$$

Where Abs_Control_ was the absorbance without sample, Abs_samples_ was the absorbance of sample extract.

### Pancreatic α-amylase inhibitory activity

The pancreatic α-amylase inhibition assay was performed according to a previous report [12]. Porcine pancreatic α-amylase (3 units/ml) was dissolved in 0.1 M phosphate buffer saline, pH 6.9. The various concentrations of the extract (10 μl) were added to a solution containing starch (1 g/l) and phosphate buffer (165 μl). The reaction was initiated by adding enzyme solution (75 μl) to the incubation medium. After 10 min incubation, the reaction was stopped by adding 250 ml dinitrosalicylic (DNS) reagent (1% 3,5-dinitrosalicylic acid, 0.2% phenol, 0.05% Na_2_SO_3_ and 1% NaOH in aqueous solution) to the reaction mixture. The mixtures were heated at 100°C for 10 min in order to stop the reaction. Thereafter, 250 μl of 40% potassium sodium tartarate solution was added to the mixtures to stabilize the color. After cooling to room temperature in a cold water bath, the absorbance was recorded at 540 nm using a microplate reader.

(2) $$\%\text{Inhibition}=\frac{Abs_{\mathrm{Control}}-Abs_{\mathrm{Sample}}}{Abs_{\mathrm{Control}}}\times 100$$

Where Abs_Control_ was the absorbance without sample, Abs_samples_ was the absorbance of sample extract.

### The intestinal α-glucosidase and pancreatic α-amylase inhibitory activities in plant-based food combinations

The combined effect of extract was determined by mixing in pairs at final concentration of 0.2 mg/ml for intestinal α-glucosidase inhibition and 2.0 mg/ml for pancreatic α-amylase inhibition. The reaction was performed according to the above assay. Results were expressed as the percentage inhibition of the corresponding control values.

### Data analysis

The IC_50_ values were calculated from plots of log concentration of inhibitor concentration versus percentage inhibition curves. The data were expressed as mean ± standard error (SE) for *n* = 3. Statistical analysis was performed by Student’s *t*-test. *P* < 0.001 was considered to be statistically significant.

## Results

### Total phenolic and flavonoid content

The list of plant-based foods used in this study is presented in Table 1. The results of total phenolic and flavonoid content of 5 plant-based foods are shown in Table 2. The total phenolic content of the extracts were in the range of 226.67-460.00 mg gallic acid equivalent/ g dried extract. Among the extracts, the highest and lowest content of total phonolic compound were observed in roselle and chrysanthemum extracts, respectively. The extracts contained flavonoid in the range of 50.3-114.8 mg quercetin equivalent/g dried extract. The highest flavonoid content was in chrysanthemum extract, whereas the lowest flavonoid content was observed in bael extract.

**Table 2:** Total phenolic compounds and flavonoids in plant-based foods

**Phytochemical analysis**		
**Samples**	**Phenolic content**	**Flavonoid content**
	**(mg/g dried extract)**	**(mg/g dried extract)**
Roselle	460.00±1.34	50.29±2.38
Chrysanthemum	226.67± 6.67	114.77±1.10
Mulberry	260.00±20.00	58.09±2.32
Bael	433.33±17.64	44.57±2.65
Butterfly pea	233.33±17.64	78.28±1.47

Results are expressed as means ± S.E.M., n = 3.

### The inhibitory effect of extracts on α-glucosidase and α-amylase activities

**Figure 1:**
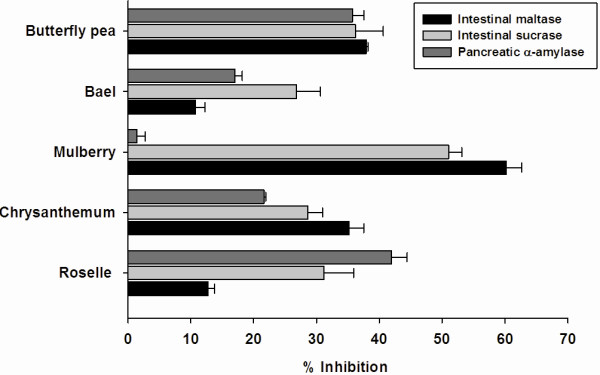
**The inhibitory effect of five plant-based foods on intestinal glucosidase (1 mg/ml) and pancreatic α-amylase (2.5 mg/ml). **The results are expressed as means ± S.E.M., n = 3.

The results in Figure 1 demonstrate the percentage inhibition of 5 plant-based foods against the intestinal α-glucosidase (maltase and sucrase). At concentration of 1 mg/ml, five plant-based foods markedly inhibited intestinal maltase, ranging from 10.8-60.28%. The intestinal maltase inhibitory activity was in the following order, from highest to lowest: mulberry > butterfly pea > chrysanthemum > roselle ≅ bael. After plotting of percent inhibition vs. log concentration of the extract, it was noted that the IC_50_ values of chrysanthemum, mulberry, and butterfly pea extracts were 4.24±0.12 mg/ml, 0.59±0.06 mg/ml, and 3.15±0.19 mg/ml, respectively (Table 3). In the meantime, 5 plant-based foods (1 mg/ml) inhibited the intestinal sucrase, ranging from 26.8-51.0%. It was found that the IC_50_ values of chrysanthemum, mulberry and butterfly pea extracts against intestinal sucrase were 3.85±0.41 mg/ml, 0.94±0.11 mg/ml, and 4.41±0.15 mg/ml, respectively. We found that roselle and bael extracts showed low α-glucosidase inhibition (the IC_50_ values > 5 mg/ml).

**Table 3:** **The IC**_**50 **_**values of plant-based foods against intestinal glucosidase (maltase and sucrase), and pancreatic α-amylase**

**IC**_**50 **_**values**			
**Samples**	**Intestinal maltase**	**Intestinal sucrase**	**Pancreatic α-amylase**
	**(mg/ml)**	**(mg/ml)**	**(mg/ml)**
Roselle	>5	>5	3.52±0.15
Chrysanthemum	4.24±0.12	3.85±0.41	>5
Mulberry	0.59±0.06	0.94±0.11	>5
Bael	>5	>5	>5
Butterfly pea	3.15±0.19	4.41±0.15	4.05±0.32

Results are expressed as means ± S.E.M., n = 3.

At the concentration of 2.5 mg/ml, the pancreatic α-amylase inhibitory activity was in the following order, from highest to lowest: roselle > butterfly pea > chrysanthemum > bael > mulberry. The IC_50_ values of roselle and butterfly pea extracts occurred at concentrations of 3.52±0.15 mg/ml, and 4.05±0.32 mg/ml, respectively. In the meantime, chrysanthemum, mulberry and bael extracts showed low α-glucosidase inhibition (the IC_50_ values > 5 mg/ml).

### The combined inhibitory effect of extracts on α-glucosidase and α-amylase activities

**Figure 2:**
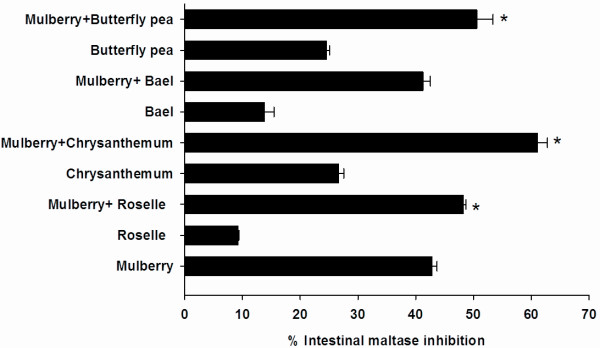
**The percentage intestinal maltase of combinatorial plant-based foods (0.2 mg/ml) with mulberry (0.2 mg/ml). **The results are expressed as means ± S.E.M., n = 3. * P < 0.001  compared to mulberry alone.

**Figure 3:**
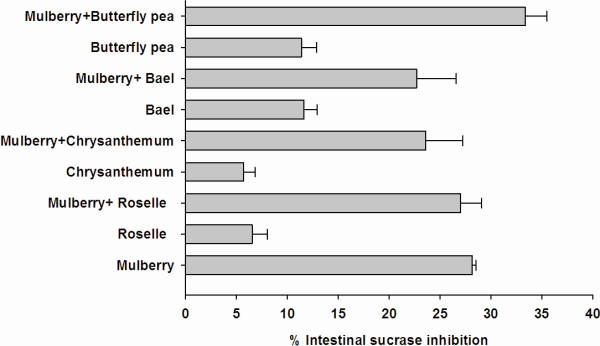
**The percentage intestinal sucrase of combinatorial plant-based foods (0.2 mg/ml) with mulberry (0.2 mg/ml). **The results are expressed as means ± S.E.M., n = 3. *P < 0.001  compared to mulberry alone.

To investigate specific combinations of plant-based foods that demonstrate synergistic or additive interactions, we selected mulberry, which showed the most potent intestinal maltase and sucrase inhibitory activities, to perform the assay as described earlier. The inhibitory effect of mulberry and its combination on intestinal maltase are shown in Figure 2. At the final concentration of 0.2 mg/ml, the percentage intestinal maltase inhibition of roselle, chrysanthemum, and butterfly pea extracts was 9.23 ± 1.73%, 26.67 ± 0.855%, and 24.52 ± 0.55%, respectively. Upon addition of mulberry extract to the solution, the percentage intestinal maltase inhibition of the mixtures significantly increased to 48.19 ± 0.53%, 61.08 ± 1.73% and 50.55 ± 2.83%, respectively. When compared with mulberry extract alone (42.84 ± 0.85%), the percentage inhibition of mixtures was nearly equal to the sum effect of mulberry and individual extract, which suggests that mulberry additively interacted with roselle, chrysanthemum, and butterfly pea on intestinal maltase inhibition. In the meanwhile, there was no increase in the percentage of intestinal maltase inhibition when bael extract was mixed with mulberry extract. The inhibitory effect of mulberry and its combination on intestinal sucrase are shown in Figure 3. When each extract (0.2 mg/ml) was added to mixture containing mulberry extract (0.2 mg/ml), it was found that there were no significant differences in the percentage inhibition of the mixtures, as compared to mulberry alone.

**Figure 4:**
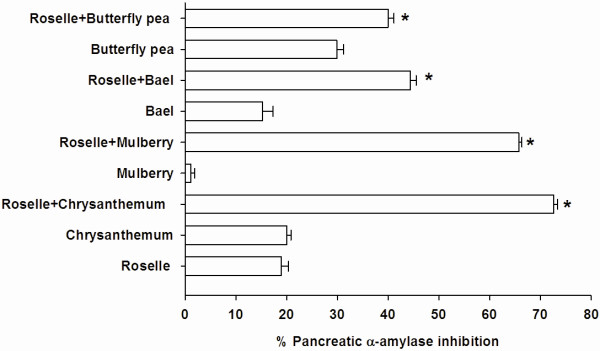
**The percentage pancreatic α-amylase of combinatorial plant-based foods (2.0 mg/ml) with roselle (2.0 mg/ml). **Results are expressed as means ± S.E.M., n = 3. *P < 0.001  compared to roselle alone.

To determine the combined effect of plant-based foods on the inhibition of pancreatic α-amylase, roselle was selected due to its demonstrating the highest inhibitory activity. Figure 4 depicts the inhibitory effect of roselle (2.0 mg/ml) and its combination with pancreatic α-amylase activity. In comparison, the individual pancreatic α-amylase activities of roselle, chrysanthemum, mulberry, bael, and butterfly pea extracts (2.0 mg/ml) were 18.99 ± 1.39%, 20.01 ± 0.86%, 1.17 ± 0.74%, 15.26 ± 2.06%, and 29.88 ± 1.34%, respectively. Significant increase in the percentage inhibition was observed when roselle extract was combined with chrysanthemum (72.60 ± 0.89%), mulberry (65.75 ± 0.60%), and bael extracts (44.40 ± 1.11%). The results indicated that the percentage inhibition of the mixtures was greater than that sum effect of individual roselle and those extracts, indicating that combination of chrysanthemum, mulberry, or bael with roselle produced synergistic inhibition against pancreatic α-amylase. When roselle extract (18.99 ± 1.39%) was combined with butterfly pea extract (29.88 ± 1.34%), the percentage inhibition (39.97 ± 1.18%) was nearly equal to the sum effect of roselle and butterfly pea extract, indicating that the combination of roselle and butterfly pea exerted additive inhibition against pancreatic α-amylase.

## Discussion

In the present study, we investigated five plant-based foods (*H. sabdariffa* (roselle); *C. indicum* (chrysanthemum); *M. alba* (mulberry); *A. marmelos* (bael); and *C. ternatea* (butterfly pea)) with anti-diabetic properties for intestinal α-glucosidase and pancreatic α-amylase inhibitory activities. The study also determined their combined inhibitory effects against intestinal α-glucosidase and pancreatic α-amylase. *H. sabdariffa* is cultivated mainly for its flowers, which are used in food applications and/or traditional medicine [13]. Chemical constituents reported in roselle flowers are phenolic compounds (protocatechuic acid), anthocyanins (cyanidin-3-glucoside), and hibiscus acid [13]. Our investigation reported that roselle extract was a potent pancreatic α-amylase inhibitor. Earlier studies indicate that the 50% aqueous methanolic extract of roselle has high inhibitory activity against pancreatic α-amylase, which is consistent with the results obtained in the present study [14]. Furthermore, the previous studies indicate that hibiscus acid and cyanidin-3-glucoside from roselle are an active pancreatic α-amylase inhibitor [15,16]. Therefore, it can be assumed that the inhibitory effect of roselle on pancreatic α-amylase may be related to these compounds.

The flower of *C. indicum* (chrysanthemum) is an herb widely used in traditional medicine in Southeast Asia for anti-inflammatory, analgesic, antipyretic purposes and the treatment of eye diseases [17]. The chemical content in *C. indicum* flower includes flavonoids, and flavonoid glycosides such as rutin, quercetin-3-glucoside, myricetin, quercitrin, and luteolin [17]. In the present study, *C. indicum* extract showed negligible pancreatic α-amylase inhibition, whereas it slightly inhibited intestinal maltase and sucrase activities.

The leaves of *M. alba* (mulberry) have been used in traditional medicine for treatment of diabetes mellitus. Recent documentation reveals that 1-deoxynojirimycin (DNJ) and its derivatives, the major component in mulberry leaves, inhibit intestinal α-glucosidases, resulting in delayed carbohydrate digestion [18]. In addition, the previous findings support the contention that administration of mulberry leaf extract significantly reduces postprandial hyperglycemia in both non-obese diabetic and healthy animals [19]. Results in this study indicated that mulberry extract had the highest inhibitory activity against intestinal α-glucosidase, whereas it had no inhibitory activity on pancreatic α-amylase. With regard to the antidiabetic effect of acarbose, the use of this drug is reported to be associated with gastrointestinal side effects caused by the excessive inhibition of pancreatic α-amylase, resulting in the abnormal bacterial fermentation of undigested carbohydrates in the large intestine [20,21]. Kwon et al. suggested that plant-based foods have lower inhibitory effect against α-amylase activity and stronger inhibitory activity against α-glucosidase, indicating that they may be effective therapeutic agents for the control of postprandial hyperglycemia with fewer side effects than acarbose [22].

*A. marmelos* (bael) is a tropical fruit native to Southeast Asian countries such as Sri Lanka, Pakistan, Bangladesh, Myanmar, and Thailand [23]. The fruits of bael are widely used in food beverage and/or traditional medicine. It has been reported that oral administration of bael fruit extract results in significant reduction in blood glucose, plasma thiobarbituric acid reactive substances, hydroperoxides, and ceruloplasmin in diabetic rats [24]. The bioactive compounds in bael fruit contain carotenoids, phenolics, alkaloids, coumarins, flavonoids, terpenoids, and other antioxidants [25].

The flower of *C. ternatea* (butterfly pea) have traditionally been used as a remedy for various diseases like urinogenital disorder, diuretic, anthelmintic, rheumatism, demulcent and anticancer [26]. Additionally, it has been scientifically studied for hypoglycemic property in alloxan-induced diabetic rats [27]. The phytochemical compounds in the flower contain delphinidin-3,5-diglucoside, delphinidin-3-glucoside, malvidin-3-glucoside, kaemferol and cyanidin [26]. In a present study, the extracts demonstrated the moderate intestinal α-glucosidase and pancreatic α-amylase inhibitory activities.

Inhibition of intestinal α-glucosidase and pancreatic α-amylase activities leads to retardation of starch hydrolysis, resulting in delayed rise in postprandial hyperglycemia [2]. It is obvious that polyphenols and flavonoids have been shown to inhibit intestinal α-glucosidase and pancreatic α-amylase *in vitro*[5-8]. Importantly, it has been reported that there is a positive relationship between the total polyphenol and flavonoid content and the ability to inhibit intestinal α-glucosidase and pancreatic α-amylase [28-30]. These correlations may explain why the inhibitory effects of plant-based food on pancreatic α-amylase and intestinal α-glucosidase may be mediated by the action of phenolic and flavonoids compounds.

To date, there have been no scientific studies on the effect of the combination of plant-based foods on inhibition of pancreatic *α*-amylase and intestinal α-glucosidase. We hypothesized that the efficacy of pancreatic *α*-amylase and intestinal α-glucosidase inhibition can be increased by the combination of these extracts. Our findings indicate that mulberry showed additive inhibition on intestinal maltase when combined with roselle, chrysanthemum and butterfly pea. It is interesting to note that roselle produced synergistic inhibition on pancreatic α-amylase when combined with mulberry, chrysanthemum and bael, whereas it showed additive inhibition when combined with butterfly pea. The study revealed that the inhibition of enzyme directly leads to an increase of hydrophilic and decrease of hydrophobic properties, leading to a failure of the formation of the active center [31]. The molecular interaction of chemical compounds in these plants on the synergistic effect remains unclear. We hypothesized that hibiscus acid and cyanidin-3-glucoside in roselle contains hydroxyl groups in the chemical structure, which can form hydrogen bonds with the polar groups in the allosteric site, situated at the entrance to the catalytic site, where other chemical compounds found in mulberry, chrysanthemum and bael bind. The results of the interactions would change the enzyme’s molecular configuration and hydrophilic and hydrophobic properties, resulting in a decrease in enzyme activities. Further study is needed to characterize the synergistic effect of chemical compounds in these extracts regarding hydrophilic and hydrophobic properties of pancreatic α-amylase.

As a consequence of the results, it is possible to increase the efficacy of intestinal maltase and pancreatic α-amylase inhibition by combination with these extracts. Based on the results, mulberry has α-glucosidase inhibitory activity, whereas roselle exhibits pancreatic α-amylase activity, which can be combined with other plant-based foods and produce the additive and synergistic interactions. The additive and synergistic effects may have positive health implications for individuals attempting to increase the inhibitory intestinal maltase and pancreatic α-amylase activities by consuming food mixtures. Current evidence supports the contention that the long-term inhibitory action of α-glucosidase inhibitors contributes to decreasing the level of HbA_1c_ in diabetic patients, resulting in a significant reduction in the incidence of chronic vascular complication such as macro- and micro-vascular diseases [32]. Consumption of combinations of plant-based food may modify *via* additive and synergistic interactions, which may help to improve postprandial hyperglycemia. This would offer a greater benefit for the treatment and prevention of diabetic and its complications. The amount of plant-based foods intake together is also required for further investigation in diabetic patients.

## Conclusion

In conclusion, the current study presents data from 5 plant-based foods evaluating the pancreatic α-amylase and intestinal α-glucosidase inhibitory activities and their additive and synergistic interactions. These results could be useful for developing functional foods that enhance intestinal α-glucosidase and pancreatic α-amylase inhibitory activities. For these reasons, further studies should focus on the outcome of investigating effects in *in vivo* activities.

## Competing interests

The authors declare that they have no competing interests.

## Authors' contributions

SA was responsible for conception and design, drafted the manuscript and revised it critically for important intellectual content. TR, PK, WS conducted the experiments, and organized the data analysis, and interpretation of data. All authors read and approved the final manuscript.

## Pre-publication history

The pre-publication history for this paper can be accessed here:

http://www.biomedcentral.com/1472-6882/12/110/prepub

## References

[B1] Gerrits PM Tsalikian E Diabetes and fructose metabolism Am J Clin Nutr 1993 58 796S 799S 8213612 10.1093/ajcn/58.5.796S

[B2] Raptis SA Dimitriadis GD Oral hypoglycemic agents: insulin secretagogues, alpha-glucosidase inhibitors and insulin sensitizers Exp Clin Endocrinol Diabetes 2001 109 Suppl 2 S265 87 10.1055/s-2001-18588 11460577

[B3] Sudhir R Mohan V Postprandial hyperglycemia in patients with type 2 diabetes mellitus Treat Endocrinol 2002 1 105 116 10.2165/00024677-200201020-00004 15765626

[B4] Hanefeld M The role of acarbose in the treatment of non-insulin-dependent diabetes mellitus J Diabetes Complications 1998 12 228 237 10.1016/S1056-8727(97)00123-2 9647342

[B5] Koh LW Wong LL Loo YY Kasapis S Huang D Evaluation of different teas against starch digestibility by mammalian glycosidases J Agric Food Chem 2010 58 148 154 10.1021/jf903011g 20050703

[B6] Pereira DF Cazarolli LH Lavado C Mengatto V Figueiredo MS Guedes A Pizzolatti MG Silva FR Effects of flavonoids on α-glucosidase activity: potential targets for glucose homeostasis Nutrition 2011 27 1161 1167 10.1016/j.nut.2011.01.008 21684120

[B7] Hargrove JL Greenspan P Hartle DK Dowd C Inhibition of aromatase and α-amylase by flavonoids and proanthocyanidins from Sorghum bicolor bran extracts J Med Food 2011 14 799 807 10.1089/jmf.2010.0143 21612457

[B8] Xiao J Kai G Ni X Yang F Chen X Interaction of natural polyphenols with α-amylase in vitro: molecular property-affinity relationship aspect Mol Biosyst 2011 7 1883 1890 10.1039/c1mb05008g 21448494

[B9] Oboh G Ademiluyi AO Faloye YM Effect of combination on the antioxidant and inhibitory properties of tropical pepper varieties against α-amylase and α-glucosidase activities in vitro J Med Food 2011 14 1152 1158 10.1089/jmf.2010.0194 21663471

[B10] Wang S Meckling KA Marcone MF Kakuda Y Tsao R Synergistic, additive, and antagonistic effects of food mixtures on total antioxidant capacities J Agric Food Chem 2011 59 960 968 10.1021/jf1040977 21222468

[B11] Adisakwattana S Lerdsuwankij O Poputtachai U Minipun A Suparpprom C Inhibitory activity of cinnamon bark species and their combination effect with acarbose against intestinal α-glucosidase and pancreatic α-amylase Plant Foods Hum Nutr 2011 66 143 148 10.1007/s11130-011-0226-4 21538147

[B12] Adisakwattana S Chanathong B Alpha-glucosidase inhibitory activity and lipid-lowering mechanisms of Moringa oleifera leaf extract Eur Rev Med Pharmacol Sci 2011 15 803 808 21780550

[B13] Carvajal-Zarrabal O Waliszewski SM Barradas-Dermitz DM Orta-Flores Z Hayward-Jones PM Nolasco-Hipólito C Angulo-Guerrero O Sánchez-Ricaño R Infanzón RM Trujillo PR The consumption of Hibiscus sabdariffa dried calyx ethanolic extract reduced lipid profile in rats Plant Foods Hum Nutr 2005 60 153 159 10.1007/s11130-005-9023-x 16395625

[B14] Hansawasdi C Kawabata J Kasai T Alpha-amylase inhibitors from roselle (Hibiscus sabdariffaLinn.) tea Biosci Biotechnol Biochem 2000 64 1041 1043 10.1271/bbb.64.1041 10879476

[B15] Hansawasdi C Kawabata J Kasai T Hibiscus acid as an inhibitor of starch digestion in the Caco-2 cell model system Biosci Biotechnol Biochem 2001 65 2087 2089 10.1271/bbb.65.2087 11676026

[B16] Akkarachiyasit S Charoenlertkul P Yibchok-Anun S Adisakwattana S Inhibitory activities of cyanidin and its glycosides and synergistic effect with acarbose against intestinal α-glucosidase and pancreatic α-amylase Int J Mol Sci 2010 11 3387 3396 10.3390/ijms11093387 20957102 PMC2956102

[B17] Wu LY Gao HZ Wang XL Ye JH Lu JL Liang YR Analysis of chemical composition of Chrysanthemum indicum flowers by GC/MS and HPLC J Med Plants Res 2010 4 421 426

[B18] Oku T Yamada M Nakamura M Sadamori N Nakamura S Inhibitory effects of extractives from leaves of Morus alba on human and rat small intestinal disaccharidase activity Br J Nutr 2006 95 933 938 10.1079/BJN20061746 16611383

[B19] Park JM Bong HY Jeong HI Kim YK Kim JY Kwon O Postprandial hypoglycemic effect of mulberry leaf in Goto-Kakizaki rats and counterpart control Wistar rats Nutr Res Pract 2009 3 272 278 10.4162/nrp.2009.3.4.272 20098579 PMC2809233

[B20] Bischoff H Puls W Krause HP Schutt H Thomas G Pharmacological properties of the novel glucosidase inhibitors BAY m 1099 (miglitol) and BAY o 1248 Diabetes Res Clin Pract 1985 1 53 62

[B21] Kwon Y-I Apostolidis E Shetty K Inhibitory potential of wine and tea against α-amylase and α-glucosidase for management of hyperglycemia linked to type 2 diabetes J Food Biochem 2008 32 15 31 10.1111/j.1745-4514.2007.00165.x

[B22] Horii S Fukasse K Matsuo T Kamed K Asano N Masui Y Synthesis and a-d-glucosidase inhibitory activity of N-substituted valiolamine derivatives as potent oral antidiabetic agents J Med Chem 1987 29 1038 1046 3519969 10.1021/jm00156a023

[B23] Baliga MS Bhat HP Pereira MM Mathias N Venkatesh P Radioprotective effects ofAegle marmelos(L.) Correa (Bael): a concise review J Altern Complement Med 2010 16 1109 1116 10.1089/acm.2009.0604 20932194

[B24] Narayan A Kumar R Kumar S Hypoglycemic and antihyperglycemic activity of Aegle marmelos seed extract in normal and diabetic rats J Ethnopharmacol 2006 107 374 379 10.1016/j.jep.2006.03.042 16781099

[B25] Sharma PC Bhatia V Bansal N Sharma A A review on Bael tree Nat Prod Rad 2007 6 171 178

[B26] Patil AP Patil VR Clitoria ternateaLinn.: An Overview Int J Pharm Res 2011 3 20 23

[B27] Sharma AK Majumdar M Some observations on the effect ofClitoria ternateaLinn. on changes in serum sugar level and small intestinal mucosal carbohydrase activities in alloxan diabetes Cal Med J 1990 87 168 171

[B28] Mai TT Thu NN Tien PG Van Chuyen N Alpha-glucosidase inhibitory and antioxidant activities of Vietnamese edible plants and their relationships with polyphenol contents J Nutr Sci Vitaminol (Tokyo) 2007 53 267 276 10.3177/jnsv.53.267 17874833

[B29] Tadera K Minami Y Takamatsu K Matsuoka T Inhibition of alpha-glucosidase and alpha-amylase by flavonoids J Nutr Sci Vitaminol (Tokyo) 2006 52 149 153 10.3177/jnsv.52.149 16802696

[B30] Ramkumar KM Thayumanavan B Palvannan T Rajaguru P Inhibitory effect of Gymnema Montanum leaves on α-glucosidase activity and α-amylase activity and their relationship with polyphenolic content Med Chem Res 2010 19 948 961 10.1007/s00044-009-9241-5

[B31] Wang Y Ma L Li Z Du Z Liu Z Qin J Wang X Huang Z Gu L Chen AS Synergetic inhibition of metal ions and genistein on alpha-glucosidase FEBS Lett 2004 576 46 50 10.1016/j.febslet.2004.08.059 15474008

[B32] Scorpiglione N Belfiglio M Carinci F Cavaliere D De Curtis A Franciosi M Mari E Sacco M Tognoni G Nicolucci A The effectiveness, safety and epidemiology of the use of acarbose in the treatment of patients with type II diabetes mellitus. A model of medicine-based evidence Eur J Clin Pharmacol 1999 55 239 249 10.1007/s002280050623 10424314

